# It’s in the mix: Reassortment of segmented viral genomes

**DOI:** 10.1371/journal.ppat.1007200

**Published:** 2018-09-13

**Authors:** Anice C. Lowen

**Affiliations:** Department of Microbiology and Immunology, Emory University School of Medicine, Atlanta, Georgia, United States of America; University of Michigan Medical School, UNITED STATES

## Introduction

**Fig 1:**
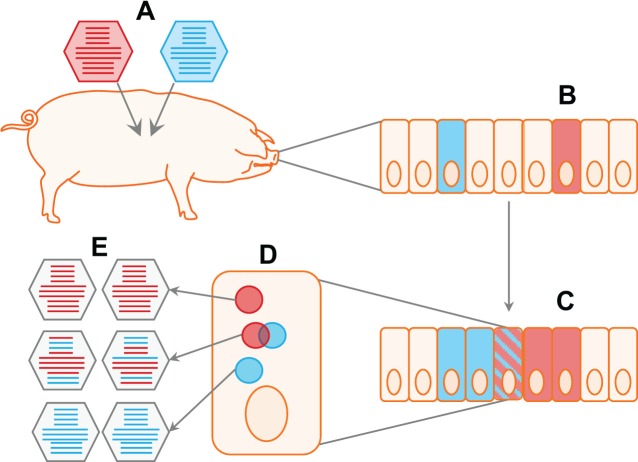
Reassortment requires viruses to meet on multiple scales. For reassortment to occur between viruses of two distinct genotypes, these viruses must infect the same host (A) and the same tissue within that host (B). Either the inoculating viruses or their progeny must come together within the same cell (C). Finally, the coinfecting viral genomes must mix within the coinfected cell, and replicated segments must be copackaged, processes which may be limited by compartmentalization of viral replication and selectivity of genome incorporation, respectively (D). When all of these criteria are met, progeny viruses of both reassortant and parental viral genotypes will emerge from the cell (E).

**Fig 2:**
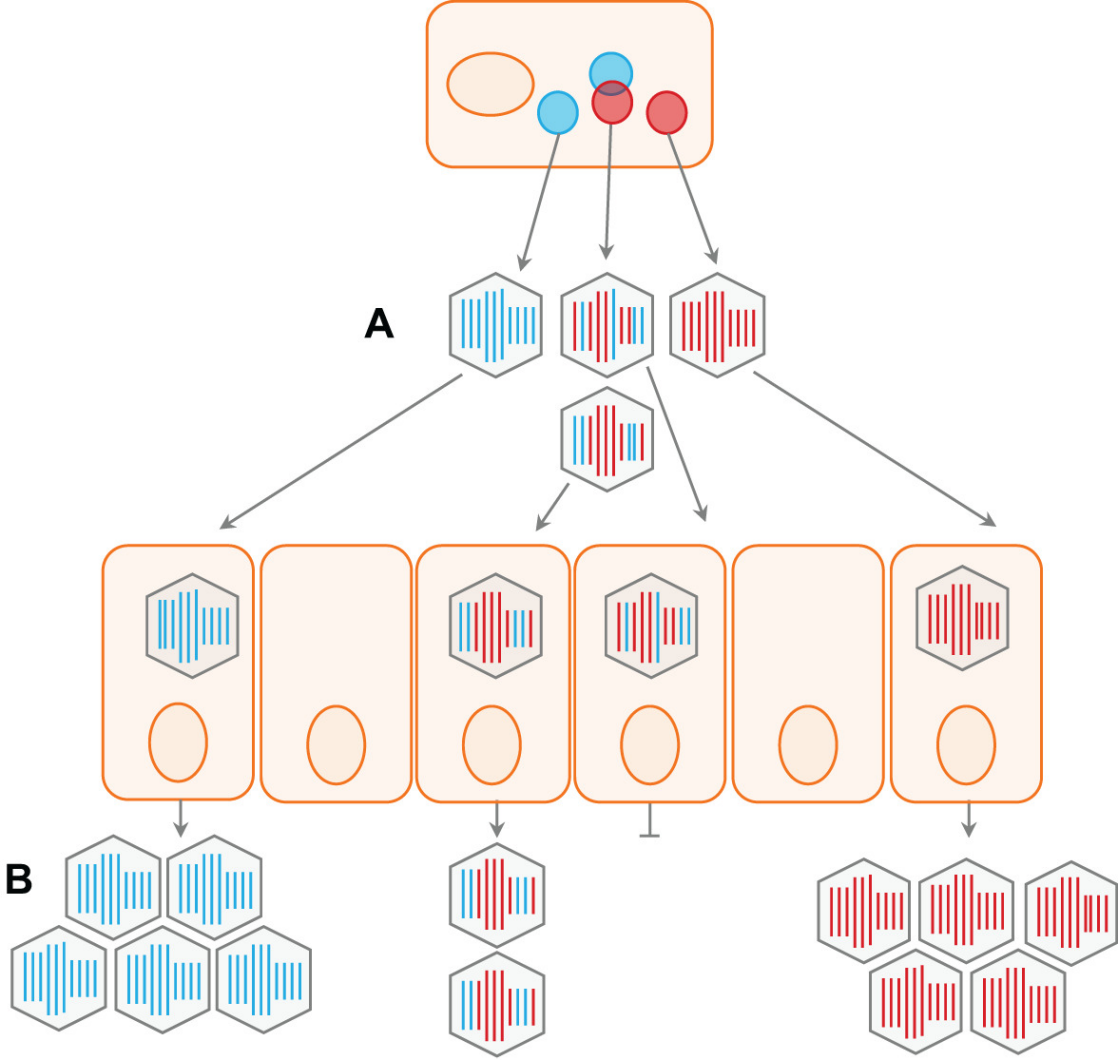
Reassortant viruses are often less fit than parental strains. The evolutionary success of reassortant progeny viruses depends on the compatibility of the reassortant genes and the selection conditions of the host environment. Thus, even when reassortment occurs efficiently (A), the prevalence of reassortant viruses may be limited by inherently low fitness and/or competition with parental viruses present in the same host or host population (B).

Segmentation of viral genomes allows exchange of intact genes between related viruses when they coinfect the same cell (Fig 1). This exchange is a type of recombination called reassortment. Classical recombination involves the joining of nucleic acid sequences derived from two different templates into one chimeric product. During reassortment, however, entire gene segments are swapped to give rise to chimeric genomes. In both cases, novel genotypes are formed, giving the potential for viruses to evolve. As with genetic change through mutation, most reassortment events yield progeny viruses that are less fit than either parent (Fig 2). Occasionally, however, reassortment gives rise to a combination of genes particularly well suited to a given set of selection pressures, and increased fitness results.

In theory, any virus with a segmented genome can undergo reassortment. Among viruses that infect vertebrates, those that carry segmented genomes belong to the Arenaviridae, Birnaviridae, Bunyavirales, Orthomyxoviridae, Picobirnaviridae, and Reoviridae. Reassortment has been documented to occur in nature for each of these viral taxa [1–6]. Nevertheless, both the frequency of reassortment and its evolutionary implications for this highly diverse set of viruses are likely to vary greatly.

## Coinfection: A necessary prerequisite for reassortment

Since reassortment takes place in coinfected cells, a critical factor governing reassortment is the frequency of coinfection (Fig 1). When thinking about a single virus population within a host, infection of individual cells with multiple viral genomes is likely to be enhanced through aggregation of virus particles and spread of virus within foci rather than dispersal throughout a tissue. In addition, if productive viral infection is fully or partially dependent on multiple infection (for example, because some viral genomes lack one or more segments), this dependency would be expected to augment reassortment. Indeed, abundant reassortment in influenza A virus (IAV) (family Orthomyxoviridae) infections occurs because fewer than eight segments are replicated in many singly infected cells [7–9]. Because all eight segments of IAV encode essential gene products, such semi-infected cells can produce progeny viruses only if the missing segments are introduced through coinfection. As a result, a high proportion of productively infected cells are coinfected [7,8]. Although not formally demonstrated to date, this phenomenon is also expected to occur for bunyaviruses (order Bunyavirales), which are thought to package less than the full complement of three genome segments into most virus particles [10,11].

Of course, reassortment has a greater impact on viral genotype if coinfecting viruses are not derived from the same population but rather represent two distinct lineages. The likelihood of such a mixed infection occurring depends on numerous factors, including prevalence of the viral lineages in circulation, likelihood of dual exposure, and the spatial dynamics of the two viruses within a coinfected host. Another important factor is the active exclusion of a second virus as a result of superinfection interference [12,13]. This phenomenon can result from direct effects of primary infection, such as viral destruction of cell surface receptors, or as a consequence of host innate immune responses, which render infected cells or an infected host refractory to further infection. Superinfection interference has been documented for diverse viruses, but it is notable that this effect appears to be minimal for certain members of the Reoviridae and Arenaviridae [14–16].

## Physical barriers to reassortment within the cell

In theory, coinfection does not necessarily lead to reassortment. The efficiency of reassortment within a coinfected cell depends on (i) the extent to which viral replication is compartmentalized within the cell and (ii) the stringency of genome packaging and compatibility of packaging signals between coinfecting viruses. The first of these factors determines the level of mixing between coinfecting viral genomes, while the second dictates whether or not segments derived from differing parental strains can be coincorporated into nascent virus particles.

Most viral life cycles are characterized by a compartmentalization of viral functions into localized areas. These can take the form of cytoplasmic inclusion bodies, viral replication organelles associated with host cell membranes, or punctate accumulations of viral components within the nucleus. The concentration of viral genetic material, viral proteins, and necessary host factors within these inclusions is thought to increase the efficiency of viral functions. However, reassortment is predicted to be limited by inclusions. If each incoming parental virus generates its own inclusion, the resultant constraint on the physical mixing of genome segments would restrict reassortment. Reoviruses (family Reoviridae) give an excellent example of a highly compartmentalized viral lifecycle. Reovirus components accumulate in distinct inclusion bodies within the cytoplasm, which are the sites of viral transcription, translation, replication, and particle assembly [17]. In this context, viral genomes replicated within the same cytoplasm may nevertheless remain unmixed. Relatively inefficient mixing of coinfecting reovirus genomes is supported by the results of experimental coinfections in which the majority of progeny viruses from coinfected cells retained a parental genotype [18]. The fact that reovirus reassortment occurs, however, is likely attributable to the merging of heterologous viral inclusions within coinfected cells (Fig 1). Reovirus inclusions are known to be dynamic, and fusion between inclusions in singly infected cells has been documented [19]. Trafficking of viral RNAs between inclusions may also be possible. In contrast to reovirus, visualization of IAV RNAs within infected cells suggested that the gene segments remain colocalized only en route to the nucleus early in infection and that the segments disperse once in the nucleus, creating the potential for mixing when multiple genomes are present [20].

Viral mechanisms that evolved to ensure the coordinated packaging of genome segments into virus particles may also place constraints on reassortment. Incorporation of viral genomes into virions is typically directed by specific nucleic acid sequences, viral protein motifs, or a combination of both. For segmented viruses, these packaging signals must be present on all gene segments. In addition, some segmented viruses enforce a high fidelity of genome packaging to ensure that one of each segment is present, a process that relies on segment-specific signals and selective incorporation. Divergence among related viruses in packaging signals may limit the potential for reassortant genotypes to form. For IAV, this form of constraint has been demonstrated experimentally [21,22]. Although the formation of viruses with heterologous packaging signals was disfavored, it was not entirely excluded. This degree of flexibility in IAV packaging gives the potential for large changes, or shifts, in viral genotype to occur when highly heterologous strains reassort.

## Fitness barriers to reassortment

In contrast to other forms of recombination, reassortment does not rely on template switching during replication and does not result in the formation of chimeric genes. Reassortment therefore does not give rise to nonfunctional genes. Nevertheless, most reassortment events are deleterious, even between similar viruses [23] (Fig 2). The reason is that many viral components act in concert, and coevolution of these components optimizes their physical or functional interactions. Reassortment, however, can abruptly pair divergent—often incompatible—viral genes. The polymerase of IAV is, for example, a tripartite complex composed of proteins encoded on three different segments. Reassortment among these segments is frequently associated with suboptimal polymerase activity and reduced fitness of the reassortant viruses [24,25]. Similarly, heterologous combinations of bunyavirus polymerase and nucleoprotein genes have been observed to be poorly compatible [26,27], and a requirement of the viral polymerase for its cognate core shell protein constrains reassortment between divergent rotaviruses (family Reoviridae) [28].

## Emergence of novel viruses through reassortment

Although usually deleterious, reassortment is very important in the evolutionary history of many segmented viruses because of the rare occasions when a reassortant virus is successful on a population scale. A striking example from the Bunyavirales is that of Ngari virus. This virus is a reassortant that derives its large and small gene segments from Bunyamwera virus and its medium gene segment from Batai virus [29]. The continued circulation of Ngari virus indicates that it is evolutionarily successful in its reservoir hosts. In addition, a marked change in virulence upon human infection has been noted: although both Bunyamwera and Batai viruses cause self-limiting febrile disease in humans, the reassortant Ngari virus has been associated with large outbreaks of hemorrhagic fever [30]. The underlying reasons for this change in virulence remain unclear. As a second example, reassortment among IAVs has been seen repeatedly to facilitate emergence into a new host niche, a phenomenon that is central to the formation of pandemic strains [31]. When an IAV adapted to a nonhuman host reassorts with a seasonal strain, resultant progeny can combine genes well adapted for replication in human cells with genes encoding hemagglutinin and neuraminidase antigenic determinants to which humans lack preexisting immunity. The antigenic novelty of such a reassortant virus gives it a significant advantage over circulating seasonal strains. If the novel virus retains or acquires the ability to replicate and transmit efficiently in humans, it can therefore spread rapidly through the population, causing a pandemic and outcompeting the previously circulating seasonal lineage.

## Prospects

Reassortment in nature is best documented for the viruses of the Orthomyxoviridae, owing to the relatively thorough surveillance and full genome sequencing efforts focused on influenza viruses circulating in human and nonhuman hosts. The extent to which reassortment contributes to emergence of novel viruses belonging to other segmented virus families is less clear, and further research is needed on this topic. In addition, the significance to virus evolution of reassortment among related variants of a single virus population is understudied. Most segmented viruses have RNA genomes and therefore have high mutation rates. Thus, even a clonal virus population is not characterized by a single sequence but is rather a cloud of related variants. By analogy to sexual reproduction in eukaryotes, reassortment within a viral population is expected to accelerate adaptive change by combinatorial shuffling of beneficial and deleterious mutations [32,33]. While homologous reassortment would not bring about the large shifts in phenotype for which reassortment is best known, it could be highly significant to viral evolution occurring over a larger timescale. Formal testing of this concept is needed to reveal the full implications of reassortment for the evolution of segmented viruses. Finally, major gaps in our understanding of reassortment lie at the mechanistic level: What processes give rise to incomplete viral genomes and consequent reliance on multiple infection? How do gene segments traverse the physical barriers imposed by viral inclusion bodies? How are heterologous segments excluded during genome assembly? Basic research aimed at addressing these questions promises to reveal important insight into the molecular processes that underlie reassortment.

## Conclusions

In summary, for viruses with segmented genomes, reassortment is an important means of genetic diversification. Its prevalence is governed by a number of stochastic effects, like the probability of dual exposure, but also by fundamental aspects of a given virus’ replication strategy. Viruses formed through reassortment of heterologous parental strains usually suffer fitness defects but occasionally possess a selective advantage and therefore can have a major impact on the evolution and epidemiology of circulating viruses.
